# Genome-wide gene expression profiling of stress response in a spinal cord clip compression injury model

**DOI:** 10.1186/1471-2164-14-583

**Published:** 2013-08-28

**Authors:** Mahmood Chamankhah, Eftekhar Eftekharpour, Soheila Karimi-Abdolrezaee, Paul C Boutros, Serban San-Marina, Michael G Fehlings

**Affiliations:** 1 Krembil Neuroscience Center, Division of Cell and Molecular Biology, Toronto Western Hospital, Toronto, ON, Canada; 2 Regenerative medicine and Spinal Cord Research Centre, Department of Physiology, University of Manitoba, Winnipeg, MB, Canada; 3 Physiology and Regenerative Medicine Program, University of Manitoba, Winnipeg, MB, Canada; 4 Informatics & Biocomputing Platform, Ontario Institute for Cancer Research, University of Toronto, Toronto, ON, Canada; 5 Department of Medical Biophysics, University of Toronto, Toronto, ON, Canada; 6 Department of Pharmacology & Toxicology, University of Toronto, Toronto, ON, Canada; 7 Biostatistix Drug Discovery/Bioinformatics, Toronto, ON, Canada; 8 Department of Surgery, Institute of Medical Sciences, University of Toronto, Toronto, ON, Canada; 9 Division of Neurosurgery and Neuroscience Program, University of Toronto, Toronto, ON, Canada

**Keywords:** Spinal cord injury, Microarray, Pathway analysis, GO enrichment

## Abstract

**Background:**

The aneurysm clip impact-compression model of spinal cord injury (SCI) is a standard injury model in animals that closely mimics the primary mechanism of most human injuries: acute impact and persisting compression. Its histo-pathological and behavioural outcomes are extensively similar to human SCI. To understand the distinct molecular events underlying this injury model we analyzed global mRNA abundance changes during the acute, subacute and chronic stages of a moderate to severe injury to the rat spinal cord.

**Results:**

Time-series expression analyses resulted in clustering of the majority of deregulated transcripts into eight statistically significant expression profiles. Systematic application of Gene Ontology (GO) enrichment pathway analysis allowed inference of biological processes participating in SCI pathology. Temporal analysis identified events specific to and common between acute, subacute and chronic time-points. Processes common to all phases of injury include blood coagulation, cellular extravasation, leukocyte cell-cell adhesion, the integrin-mediated signaling pathway, cytokine production and secretion, neutrophil chemotaxis, phagocytosis, response to hypoxia and reactive oxygen species, angiogenesis, apoptosis, inflammatory processes and ossification. Importantly, various elements of adaptive and induced innate immune responses span, not only the acute and subacute phases, but also persist throughout the chronic phase of SCI. Induced innate responses, such as Toll-like receptor signaling, are more active during the acute phase but persist throughout the chronic phase. However, adaptive immune response processes such as B and T cell activation, proliferation, and migration, T cell differentiation, B and T cell receptor-mediated signaling, and B cell- and immunoglobulin-mediated immune response become more significant during the chronic phase.

**Conclusions:**

This analysis showed that, surprisingly, the diverse series of molecular events that occur in the acute and subacute stages persist into the chronic stage of SCI. The strong agreement between our results and previous findings suggest that our analytical approach will be useful in revealing other biological processes and genes contributing to SCI pathology.

## Background

Human spinal cord injury (SCI), often the result of both impact and varying degrees of compression, is initially a primary mechanical tissue and cell injury, but further develops into a cascade of complex secondary damage [1]. Accordingly, the need for biologically relevant animal SCI models has focussed on the development of animal injury models that can reliably mimic human SCI [2]. Various animal SCI models can be classified based on how the primary injury is induced (either physical or chemical), and the duration and extent of the primary injury. Techniques such as weight drop, clip compression, calibrated forceps and chemically-mediated SCI have been introduced and evaluated in laboratory animal models [3-5]. The majority of primary injuries in animal SCI models are physically-induced, by either impact, compression, or a combination of both; the latter most closely mimic SCI in human patients. The nature of the primary injury will dictate the types of secondary events that contribute to common outcomes of all injury models such as acute and chronic spinal cord dysfunction [6] and loss of regenerative capacity [7,8]. It may also contribute towards unique features and characteristics of each injury model such as spasticity [9,10], neuropathic pain [11,12] and systemic effects [13]. Finally, various methods and devices can be calibrated to injure the spinal cord for various durations of time; hence, the primary injury can be classified as transient or persistent.

Amongst the injury models, the weight drop [14-19] and the aneurysm clip [20-23] are the most standard graded methods of physically-inducing experimental SCI, which have been thoroughly characterized in laboratory animal models. In weight drop models [14-19], the primary injury is a transient impact and compression, hence the name contusive injury, which can be graded as mild, moderate or severe depending on the weight and height of the drop. The clip compression model was introduced as one of the first non-transection models of SCI in rodents [20]. It is an easy and highly reproducible injury model and has the ability to mimic different levels of injury by adjusting the force and duration of clip application. The method of primary injury in the clip model is slightly different from the weight drop model as the compressive force due to the closure of the clip is maintained on the spinal cord for a defined period of time. Consequently, the outcome of a clip injury is usually a more severe form of vascular network disruption, which leads to hemorrhage and shortage of blood supply to the tissue rather than a contusive injury.

Various SCI injury models have been characterized by examining the primary injury (impact, compression, contusion, or laceration of the tissue) and the secondary injuries (blood-spinal cord barrier (BSCB) permeability, ischemia, edema, apoptosis, glutamate excitotoxicity, inflammation, demyelination, axonal degeneration, reactive gliosis, and scar tissue formation) to the spinal cord tissue using low- and high-resolution microscopy and immunohistochemical methods [24,25]. Additionally, the extent of damage and functional recovery in animals is recorded using kinematic and behavioural studies [26,27]. Studying the functional state of neurons after injury or during the recovery process is another approach but is only feasible using electrophysiological methods [28].

Our lab has successfully used the clip compression injury model to injure the rat spinal cord at the thoracic level with consistent and reliable results; both acute and chronic SCI in rats have been characterized using this model [21-23,29-32], as well as assessment of the efficiency of various intervention strategies such as a combination transplantation of mouse brain-derived neural precursor stem cells, chondroitinase, and growth factors [6,29,30,32-35]. However, molecular events following clip compression injury have not been explored using high throughput strategies. In this study, we used the Affymetrix GeneChip Rat Genome 230 2.0 platform for microarray gene expression analysis of SCI using the clip compression injury model in rats. A unique feature of this study is that a more comprehensive catalogue of the whole genome transcript levels was compared across a wider time frame, i.e. 1, 3, 7, 14 and 56 days post-injury, than has been examined in previous work. In this study, we present the overall picture of biological processes that relate to stress response and are up-regulated and the corresponding molecular events. We show that, by systematically applying the controlled vocabulary of gene functions presented in Gene Ontology (GO) domains, the temporal pattern of biological processes are extracted from microarray gene expression data and that this approach can be applied to discover novel molecular events.

## Results

### Feature analysis of affymetrix GeneChip Rat genome 230 2.0 array

Analysis and filtering on the resulting file of 31,042 ProbeSets revealed that 10,791 ProbeSet IDs had no annotations, i.e. no Entrez IDs or official gene symbols, which were flagged out. This reduced the number of workable ProbeSet IDs to 20,251. In addition, there were duplicate or multiple ProbeSet IDs which represented a single gene. Conversely, there were ProbeSet IDs with multiple annotations (EntrezID/Gene Symbol) due to sequence identity across more than one gene segment in the genome. This issue could not be easily resolved as the level of uniqueness of the oligonucleotide sequence is not high enough to allow annotation to one gene exclusively. This feature requires manual curation of the data based on Affymetrix instructions to use the latest annotation, which is also the most relevant. Taking the above two features into consideration results in 14,324 gene symbols on the GeneChip RG230 2.0 array. The resulting data file still contains the ProbeSet IDs that have “LOC” or “RGD” identifiers instead of actual gene symbols. These identifiers are applied to genes that are less well characterized and usually belong to similar or orthologous proteins in other species. They may also belong to non-coding regions of the genome. Software platforms developed for GO enrichment and pathway analysis rarely map the LOC and RGD identifiers. In the original Affymetrix GeneChip 230 2.0 array annotation file, the number of LOC and RGD annotated ProbeSets sets are 1163 and 1135, respectively. The same issue of duplicate/multiple entries also applies to these ProbeSets, hence the numbers of “LOC” and “RGD” identifiers in the final output file with 14,324 entries were less: 939 for the LOC and 829 for the RGD identifiers. This means that the total number of annotated ProbeSets in the Affymetrix GeneChip Rat Genome 230 2.0 array annotation file that were mapped to known gene candidates was 12,557, which is equivalent to 62.4% and 71.2% of the total number of genes annotated and listed in the Rat Genome Database (RGD) and European Bioinformatics Institute (EBI) association files, respectively.

### Analysis of ProbeSet data

**Figure 1:**
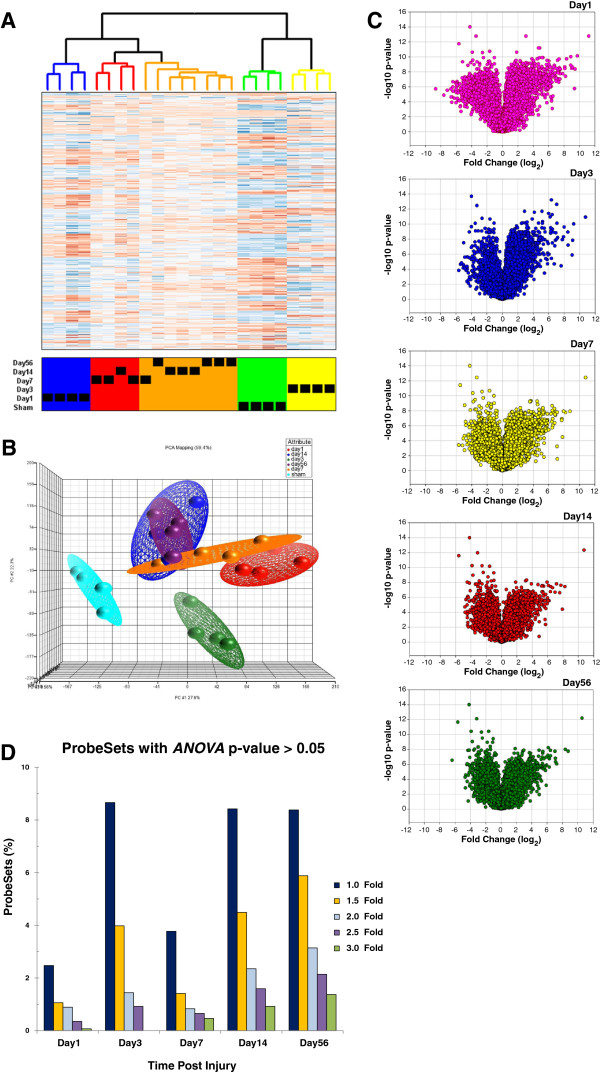
**Time - Point ProbeSet Data Analysis. A**. Unsupervised machine learning grouping of animals by expression. To visualize temporal patterns as well as inter-animal variability, unsupervised machine learning was employed followed by a divisive hierarchical clustering algorithm (DIANA) to cluster differentially expressed ProbeSets in any pair-wise contrast (see Methods). Finally, standard agglomerative hierarchical clustering was used to group animals. The result is visualized using the Heatplus package of BioConductor. Heatmap (columns: samples; rows: genes, in red and blue coloring, depicting up- and down-regulation respectively). **B**. Principal Component Analysis of Individual Time Point Transcripts. Using Partek GS version 6.5, we performed principal component analysis (PCA) of the 33042 transcripts on the 230 2.0 GeneChip array for all animals at each time point to assess variability of the data across individual animals and time points. There are inter-individual differences but eclipses show that there are no outliers in our experiment. Additionally, the eclipses of Day 7, 14 and 56 cross each other, which indicate some level of commonality between these time points, as was evidenced and shown in the tree view of the heat map. **C**. Volcano plots of fold change values of all 33042 ProbeSets vs. transformed (− log_10_) ANOVA *t* test p-values. Individual time point data were plotted for comparison. ANOVA *t* test p-values for pair-wise contrasts between each time point data relative to sham were calculated and transformed to - log_10_ values and plotted against fold change values. **D**. Percentage of ProbeSets with ANOVA *t* test p-values higher than 0.05. The percentage of ProbeSets with p-values higher than 0.05 was calculated at all time-points and plotted at various fold change values.

We used a divisive hierarchical clustering algorithm (DIANA) to identify the strongest trends within the dataset, in all pair-wise comparisons (see Methods). The results were visualized using the Heatplus package of BioConductor (Figure 1A). Interestingly, three main clusters were observed, corresponding to the sham controls (green cluster), day 1 animals (blue cluster), and day 3 animals (yellow cluster). The later time-points are further subdivided into a cluster composed primarily of day 7 animals (red cluster) and another composed primarily of day 14 and day 56 animals (brown cluster). Principal component analysis of the ProbeSet data was performed to assess variability between individual animals in each group and also at different time points and resulted in clustering of the transcripts belonging to each group of sham or injured animals shown in Figure 1B. There are inter-individual differences but eclipses show that there are no outliers in our experiment. Additionally, the eclipses of day 7, day 14 and day 56 cross each other, which indicate some level of commonality between these time points, as was evidenced and shown in the tree view of the heat map.

Data normalization and expression/signal value determination resulted in a list of all 31,099 ProbeSets, their fold change values relative to sham (in Log_2_ scale), and associated ANOVA *t* test p-values across the time points. Volcano plots of the corresponding fold change values against transformed (−log_10_) p-values for every time point are displayed in Figure 1C. As shown, all volcano plots display a normal distribution of ProbeSets with fold change values from −8.7 to 11.2 for down- and up-regulated genes, respectively. The shape of the volcano plot changes as time post-injury goes by. Thus, day 3 ProbeSet data plots are not as populated, especially on the down-regulated area and are less similar to other data points. The day 1 plot, on the other hand, looks more similar to the day 7 volcano plot. The more chronic data points of day 14 and day 56 look more similar to each other than to earlier data points.

Examination of the number of ProbeSets with marginal ANOVA *t* test p-values gave an estimate as to the reliability of data obtained. Thus, we analyzed our data for the number of ProbeSets with ANOVA *t* test p-values higher than 0.05 at different fold change values (Figure 1D). We found that the majority of changes in gene expression with significance levels of p > 0.05 generally belong to ProbeSets with lower fold change values. For example, the number of ProbeSets with ANOVA *t* test p > 0.05 did not exceed 6% of the total number of ProbeSets, irrespective of the fold change values. At a more stringent significance level of p ≤ 0.001, however, it would be necessary to filter out the ProbeSets with expression values less than 2 fold changes in order to keep the number of filtered ProbeSets around 10% or less across the time points (data not shown). Thus, filtering the data with higher fold change values automatically targets for transcripts with smaller *t* test p-values. Based on the results presented in Figure 1D, we performed the functional analysis on the ProbeSet data with fold change values of  ≥ 1.5 and p ≤ 0.05.

### Analysis of gene set data

To explore our data at gene level, additional analysis and filtering was performed on the resulting file of 31,042 ProbeSets as mentioned earlier. In order to finalize the gene set data at different time-points for functional analysis, those transcripts with ANOVA *t* test p-values ≥ 0.05 were removed from the initial list and the resulting data were analyzed using STEM so that fold change values for genes with multiple ProbeSets are averaged based on the median values. Table 1 shows the results of this analysis by listing the number of deregulated transcripts at each time-point and at different fold change values (p ≤ 0.05). For example, on day 1 post-injury, there are 2,500 transcripts with at least 1.5 fold changes in expression level. This number diminishes significantly on the following days to about half, but nevertheless, stays at a significantly high value, more than 1,000 transcripts, even at 8 weeks post-injury (Table 1). The majority of gene expression changes are up-regulations especially on day 1 post-injury. Although the total number of deregulated transcripts is reduced to about 1,304 on day 3, the ratio of up- vs. down-regulations is increased significantly (1.9 on day 3 compared to 1.5 on day 1). Between day 7 and day 56 post-injury, changes in the spinal cord transcriptome tend to approach a steady state. The finding that variations in the number and level of transcripts’ deregulations were highest during day 1 and day 3 is consistent with the fact that most signaling responses to the damage incurred by mechanical impact and compression are communicated within the hyperacute and acute stages of the trauma.

**Table 1:** Time-point gene set data analysis at different fold change criteria (p ≤ 0.05)

**Time-points**	**≥ 1.0 fold change**	**≥ 1.5 fold change**	**≥ 2.0 fold change**
**Day1**			
Up	2430	1511	972
Down	1579	989	620
** Total**	**4009**	**2500**	**1592**
**Day3**			
Up	1687	864	502
Down	940	440	208
** Total**	**2627**	**1304**	**710**
**Day7**			
Up	1593	922	615
Down	1062	581	322
** Total**	**2655**	**1503**	**937**
**Day14**			
Up	1126	696	479
Down	1056	577	327
** Total**	**2182**	**1273**	**806**
**Day56**			
Up	1173	729	482
Down	995	537	284
** Total**	**2168**	**1266**	**766**

Transcripts with ANOVA *t* test p-values ≥0.05 were removed from the initial list and the resulting data were analyzed using STEM to average the fold change values based on the median of fold changes for genes with multiple probe sets. ProbeSets with different fold change values combined with ANOVA *t* test p-values ≤0.05 were considered for this analysis.

**Figure 2:**
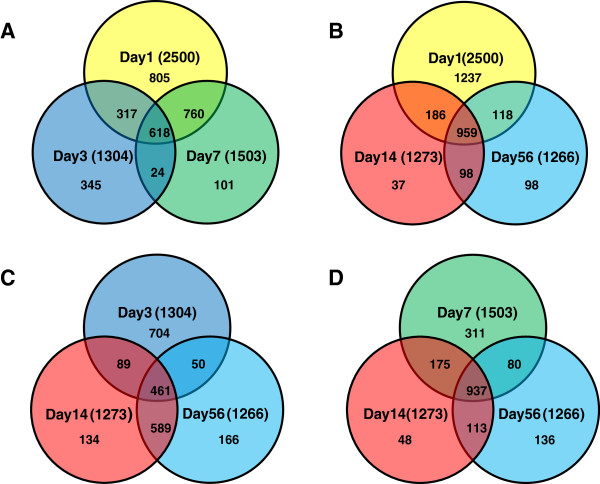
**Time-Point Gene Set Data Analysis. A-D**. Relationship between the nature of deregulated transcripts at different time points. Deregulated transcripts (fold change ≥ 1.5) at each time point were examined for common and unique genes using a Venn diagram. Overlapping areas represent common genes between different time points. Day 1 deregulated genes were compared with day 3 and day 7 transcripts in **A**, and with day 14 and 56 in **B**. Day 3 and Day 7 deregulations were compared with both day 14 and day 56 in **C** and **D**, respectively. The transcriptome on day 1 is more similar to day 7 and less similar to days 3, 14 or 56. Additionally, day 14 and 56 deregulations are the most similar to each other with about 82% of the genes common between the two time points.

We next examined the nature of deregulated transcripts at different time-points relative to each other. Figure 2 (A-D) depicts the Venn diagrams with overlapping regions demonstrating the number of common genes showing changes at various time points as well as time-point specific genes. In terms of gene contents, the day 1 pattern of gene expression is more similar to day 7 as is evidenced by 760 common genes between day 1 and day 7, compared to 317 between day 1 and day 3, 186 between day 1 and day 14 and 113 between day 1 and day 56. On the other hand, each time-point has unique genes, whose expressions do not appear to change at other time points. This observation supports the notion that, although some processes that are invoked early after SCI may stay active throughout the acute or chronic phase, there are unique features to the early response genes that are dramatically different from the response in the following days or weeks post-injury. Additionally, deregulated transcripts on day 14 and day 56 were found to be very similar to each other with approximately 82% of the genes showing changed expression being identical at these two time-points. This result was also predicted from the heat map (Figure 1A). This indicates that the biological processes in response during the chronic phase of SCI remain constant.

### Time-series expression profile clustering by STEM

As our data were collected at different time-points, we performed time-series expression profile clustering to search for common temporal expression patterns. To allow clustering at a reasonable number of possible model profiles, the parameter for “STEM clustering method”, “model profiles” was set to 50 and 2 was selected as the “maximum unit change between time points”. To facilitate interpretation of our data in the context of previous microarray studies, we used a cut-off of 1.5 fold change (up and down) as has been previously reported [36-38]. Additional file 1: Figure S1 depicts the results of the 50 expression profiles obtained with STEM, at 1.5 fold change benchmark value relative to sham controls. The profiles are shown in decreasing order of significance of clustering by STEM, from the lowest to the highest p-values. Eight expression profiles were statistically significantly enriched relative to the number of genes that would occur in these profiles by chance alone. As shown, the corrected p-values range from the lowest for profile 44 to the highest for profile 2. Table 2 summarizes the number of significantly deregulated transcripts across all time-points with respect to the two criteria of “Maximum Number of Missing Values” and “Minimum Absolute Expression Change (from Zero)”. As shown, at the most stringent condition of “zero” missing values, 1,251 genes pass the filtering criteria of 1.5 fold change, of which 1,074 genes (86%) were clustered in the 8 expression profiles and the remaining 177 genes (14%) were assigned to other non-significant profiles. We performed our time-series analysis allowing 1 missing value (Additional file 1: Figure S1). This resulted in 2,058 genes passing the filtering criteria with 85% of deregulated transcripts assigned to eight expression profiles 44, 6, 46, 1, 0, 48, 41 and 45.

**Table 2:** Time-series gene set data analysis by STEM at different fold change criteria (p ≤ 0.05)

**No. missing values allowed**	**≥ 1.0 fold change**	**≥ 1.5 fold change**	**≥ 2.0 fold change**
0	1820	1251	831
1	3066	2058	1367
2	3916	2507	1615
3	4577	2848	1829
4	5050	3087	1958

Fold change values with ANOVA *t* test p-values ≥0.05 were removed from the initial list and the resulting data were analyzed using STEM for time-series expression analysis at different values for the “Maximum Number of Missing Values” parameter.

**Figure 3:**
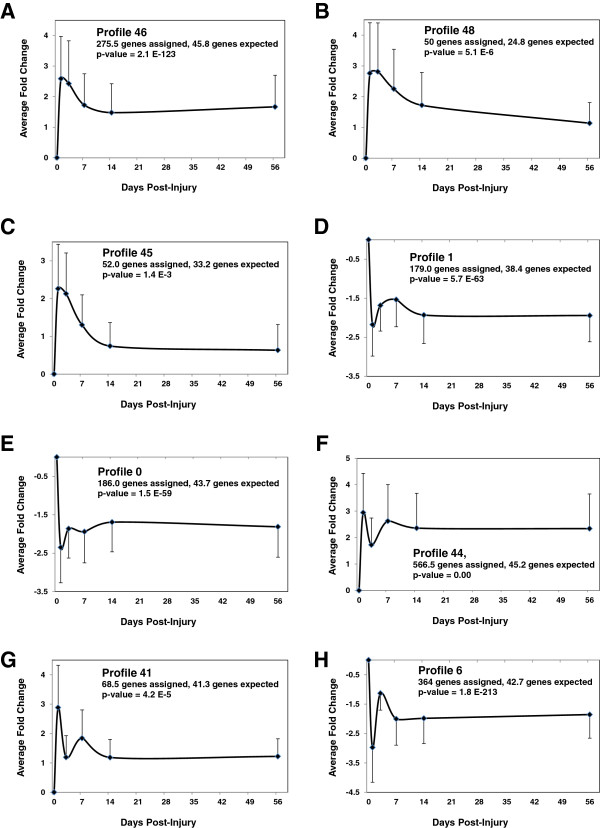
**Distinct Significant Expression Profiles Clustered by STEM. A-H**. Average fold change values for the genes in each cluster were plotted against the real time scale of the time-points post-injury. Error bars denote the standard deviation of the mean. Three classes of expression profiles are observed. Class I profiles **(A**-**C)** display an “up-down” pattern with the peak of up-regulation in 24 hours (profiles 45, 46 and 48) post-injury. The up-regulation is followed by decline of transcript levels, either sharply back to normal values (profiles 45 and 48) or gradually to higher than control values (profiles 46). Class II profiles (1 and 0) are quite similar to each other as they display down-regulations of many genes on day 1, which stay at lower than normal levels even at 8 weeks post-injury. Class III profiles **(E**-**H)** represent fluctuating profiles and are subdivided into two clusters. Cluster I (Profiles 44 and 41) is marked by an early increase in gene expression by day 1 followed by sudden decline in transcript level at day 3. In profile 44, this transient change in transcription level is followed by an escalating condition whereby the same transcripts are again up-regulated by day 7 and stay at higher than control values until 8 weeks post-injury. In cluster II (profile 6), a reverse phenomenon is observed, where the early event is a sharp decrease in transcript level and a follow up fluctuation pattern in gene expression. Despite fluctuations in gene expression levels, the transcript levels of genes in profiles 6 remain significantly lower than control levels throughout the course of experiment.

To simplify the graphical presentation of the data, fold changes in expression values for all genes associated with only the statistically significant profiles were averaged and plotted against the post-injury observation time points (Figure 3A-H). Two broad profile classes become apparent from these data: "up-down/down-up genes” (Figures 3A-E) and “fluctuating genes” (Figures 3F-H). The “up-down” category comprises the cluster of profiles that share a pattern of up-regulation early after injury over the course of 24 hours (profiles 45, 46 and 48). In these profiles, the early response is subsequently followed by down-regulation of the genes clustered in these profiles. This occurs gradually, approaching a steady level at normal or higher than normal values. Although genes in profiles 45 and 48 were allocated to two separate expression profiles by STEM, visual examination indicates striking similarities between the two profiles. Patterns of late phase expression in these profiles look similar to each other as the late expression values of almost all gene transcripts in both profiles approach values comparable to those of the sham animals. For many genes in profile 46 the pattern is different as the transcript levels of about 50% of genes in this profile remain up-regulated throughout the course of the study and also at the end of 8 weeks, hence their functions seem essential not only in the acute and subacute phase but also during the chronic stage of the injury. Profiles 1 and 0 are quite similar to each other as they display down-regulations of many genes on day 1, which stay at lower than normal levels even 8 weeks after injury.

Class II profiles represent fluctuating profiles (44, 41 and 6), with a surprising but more complex pattern of gene expression, most notably during the 24–72 hours post-injury (Figure 3G-H). This results in a bi-phasic expression pattern, which falls into two main clusters. The first cluster comprises profiles 44 and 41 (Figure 3F-G) and is characterized by an initial up-regulation of gene transcript levels early on day 1, followed by a sharp decrease in gene expression on day 3. More than 53% and 83% of the genes in profiles 44 and 41, respectfully, displayed at least a 1.0 (Log_2_ scale) fold change reduction in transcript levels on day 3 compared to day 1. For profiles 44 and 41, this bi-phasic pattern of gene expression is further followed by escalation of gene expression, which peaks at day 7 and stabilizes on day 14 onward. The second cluster only includes profile 6, which is essentially the mirror of profile 44 and comprise down-regulated genes (Figure 3H). It is characterized by an early and substantial down-regulation on day 1. Next, a period of recovery to normal transcript levels is observed that peaks on day 3 post-injury and then switches direction again and remains low through today 56. Finally, detailed information in Figure 3 indicates that the majority of transcripts belong to profile 44 and 6 with up-regulated transcripts clustering in the former and down-regulated transcripts in the latter.

In summary, the following conclusions can be drawn from the cluster analysis of transcripts both at the ProbeSet and gene level following clip-compression injury of the spinal cord in rats:

– Major molecular events after introduction of clip-compression injury occur immediately and up to 72 hours post-injury

– For many transcripts a bi-phasic pattern of gene expression is observed, possibly due to switching mechanisms acting between day 1 and day 3 or a shift in the cellular origin of deregulated transcripts or the type of response elicited resulting in chronic deregulations of many genes. Therefore, for many transcripts, the late up or down-regulations seem to be distinct from the early response

– The early events seem to stabilize for most transcripts by 1 week post-injury, i.e. no more dramatic global changes in the average gene expression are observed and the level of expressions remains relatively constant.

### GO enrichment analysis of deregulated genes

#### *Choice of reference association file*

Gene Ontology (GO) enrichment analysis was preferred as the method of choice for functional analysis of the list of deregulated genes as it is based on a controlled vocabulary of terms at all three domains of “Biological Process” (BP), “Molecular Function” (MF) and “Cellular Compartment” (CC). Initially, gene association files from RGD or EBI were analyzed for the number of rat genes that are annotated at each of the three domains (BP, MF and CC) and compared with the list of significantly (ANOVA *t* test p ≤ 0.05) deregulated genes (Fold Change ≥ 1.0 and 1.5) at each time point. We found that about 70-75% of deregulated transcripts were annotated for all three domains of GO, in reference to the RGD association file whereas the association file from EBI only annotated 55-65% (data not shown). This implies that a minimum of 25-30% of significantly deregulated transcripts are not annotated (in any BP, MF or CC domains) in any gene ontology association files and thus are not considered for analysis regardless of the type of software platform used to perform GO enrichment analysis. Therefore, due to its more extensive annotation coverage, GO enrichment analysis in this study was performed in reference to the RGD association file.

#### *Fold change and p-value criteria affect the number of enriched terms*

**Figure 4:**
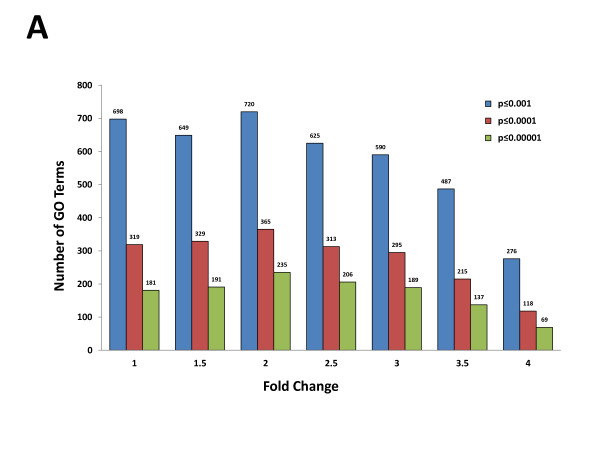
**Time-Series Gene Ontology (GO) Enrichment Analysis of Deregulated Transcripts after SCI. A**. Number of enriched GO terms as a function of fold change and p-value of enrichment parameters. Filtering criteria in STEM were set to different values between 1 and 4, and the number of Biological Process GO terms with corrected p-values of ≤ 0.001, ≤0.0001 and ≤ 0.00001 were calculated and plotted. **B**. Distribution of enriched GO terms with to GO levels 3–20. GO enrichment analysis was performed on transcripts with a minimum of 1.5 fold changes in expression (ANOVA *t* test p ≤ 0.05). This resulted in 329 and 649 enriched GO terms at p-value cutoffs of 0.0001 and 0.001, respectively. Enriched Biological Process GO categories were positioned on the directed acyclic graph (DAG) structure of the gene ontology hierarchy. The number of significant GO terms at every GO level at were plotted and also shown on top of each bar. Note that at p ≤ 0.001, no GO terms were obtained at GO levels 15–17.

GO enrichment analysis can result in numerous enriched GO terms with overlapping or redundant terms making the reduction and prioritization task difficult. Depending on the fold change value criteria, which determine the number of deregulated transcripts, the number of enriched GO terms can vary. In order to rationalize an approach where meaningful numbers of GO terms are achieved, we first examined how the parameter of fold-change in expression can affect the number of enriched terms. We chose to perform this preliminary analysis in a time-series fashion, meaning that deregulated transcripts with significant fold change values (ANOVA *t* test p ≤ 0.05) across all time-points were considered and only one missing value was permitted. Thus, deregulated transcripts at 1–4 fold change values in at least one time point were separately subjected to GO Biological Process (BP) enrichment analysis (minimum GO level of 3, minimum number of genes of 5) and the number of GO terms were plotted as a function of fold change in expression (Figure 4A). As shown, this time-series analysis at 1.0, 1.5 and 2.0 fold change values resulted in very high numbers of enriched terms, e.g. 698, 649 and 720 GO terms (with the adjusted p-values ≤ 0.001), respectively. Setting the fold change criteria to higher values did not limit the number of GO terms as it only gradually declined (Figure 4A). For example, GO enrichment analysis on the list of transcripts with 2.5, 3.0, 3.5 and 4.0 fold change values resulted in 625, 590, 487 and 276 terms (p ≤ 0.001), respectively. It is interesting to note that significant reduction in the number of GO terms is not achieved until much higher fold change values are considered, e.g. 4 fold in Log_2_ scale (64 fold in normal scale), which also significantly reduces the number of deregulated transcripts included in the analysis (data not shown). However, examining the number of terms at lower p-values remarkably reduced the number of GO terms, although similar trends across different fold change values were observed (Figure 4A). For example, changing the p-value parameter from 10^-3^ to 10^-4^ reduced the number of enriched GO terms to about half or even less, at all fold change values (Figure 4B). Therefore, it seems that the logical approach for enrichment analysis in regard to the fold change and p-value criteria is to include all transcripts with even lower fold change values to include higher number of genes and especially to avoid excluding potential key regulatory genes in the analysis, which may not have displayed dramatic changes in expression. Finally, although the actual size p-value cutoff seems to be a much more important parameter in limiting the number of enriched GO terms to a workable value, it may not help with prioritization of the enriched terms and their specificity.

#### *GO level criteria and term specificity*

Gene ontology hierarchy consists of a tree of inter-related terms in a distinct structure called a directed acyclic graph (DAG). In GO tree hierarchy, the terms Biological Process, Molecular Function, and Cellular Component are at level 1. Therefore, more general parent terms are at the top of the hierarchy with lower GO level values and higher GO level values are assigned to more specific child terms. Unless more than one parent is assigned, GO level can be considered as a constant value for each term. As GO level values refer to the position of the enriched terms in the GO hierarchy tree, they can define the specificity or granularity of a given GO term and thus are a valuable parameter for terms prioritization and for inferring biological meaning from GO enrichment analysis [39]. To determine the position of each of the enriched GO terms in the DAG structure of the gene ontology hierarchy, we performed GO Biological Process (BP) enrichment at GO levels between 20 and 3, using STEM. This led to multiple lists of enriched and overlapping GO terms at each level of GO hierarchy. Using this approach, a single GO level was assigned to every GO term. Figure 4B depicts the distribution of all 649 and 329 GO terms obtained at p ≤ 0.001 and p ≤ 0.0001 cut offs, respectively, against their corresponding GO levels. As shown, the enriched terms show a distribution curve that is close to normal against different GO levels though it is slightly skewed at the higher GO level value side. The majority of terms were obtained when GO level parameter was set to 11 and less. On the other hand, examining levels lower than 5 led to GO terms with lower p-values at the cost of more general terms with much broader information about the function of genes in that category (data not shown). It should be mentioned that, although more general terms offer less specific information about the actual biological functions of deregulated transcripts in the list, their significance level, marked by their p-value of enrichment, along with their GO level can help delineate how the specific terms are related to the correct parental signaling pathways or biological processes.

#### *Time-series vs. Time-point analysis*

Temporal analysis of gene expression may imply analysis of gene lists in either a time-series and/or a time-point fashion. Although STEM has been designed for time-series expression profiling prior to GO enrichment, it can also be used for time-point GO enrichment analysis. In the time-series approach, clustering by STEM produces significant expression profiles followed by enrichment analysis of the list of genes in each expression profile. The complication with time-series analysis is that not all transcripts have accepted ANOVA *t* test p-values (e.g. p ≤ 0.05) and thus the insignificant expression values must be removed from the original data prior to STEM analysis. To resolve the issue of many transcripts with missing values across all time-points, STEM offers the option to set the missing value parameter. However, depending on the selected value, this may ultimately reduce the total number of deregulated genes included in the functional analysis. In the time-point approach, however, the input file is the list of genes that belong to a specific time-point, in which case the number of missing values is not an issue. In this study, the time-point GO enrichment analysis was employed to discover common up- and down-regulated biological processes across the time-points as well as possible unique processes to each time-point. The output GO terms were used for inter-relationship analysis and or visualized as a scatter plot or interactive graph using REViGO [40].

#### *Time-series GO enrichment*

**Figure 5:**
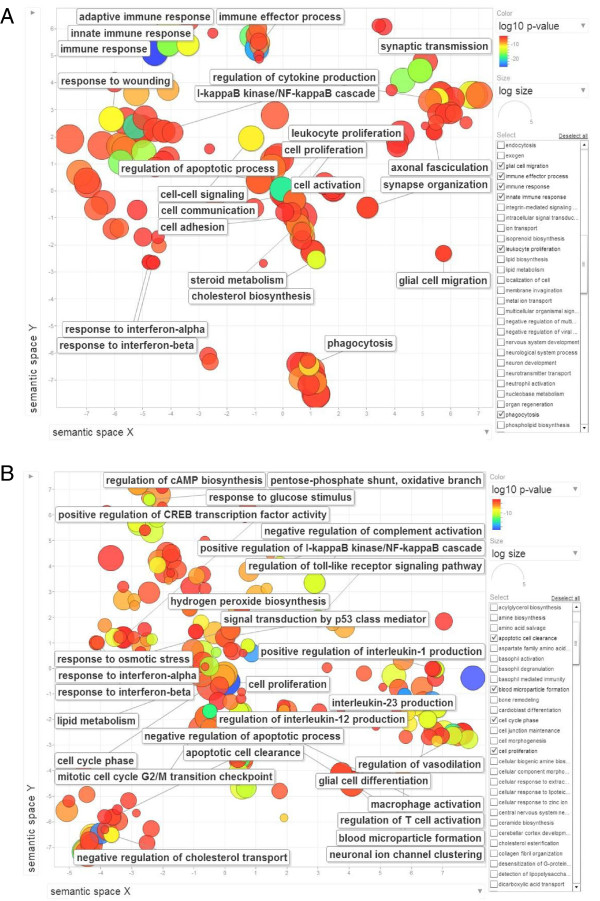
**REViGO Scatterplot of the Enriched GO Cluster Representatives from Time-Series Analysis.** Time-series GO enrichment analysis for transcripts with a minimum of 1.5 fold change regardless of their expression profile at p-value cut off of 0.0001 led to 329 GO terms at GO level ≥3 and 267 terms at GO level ≥5. The resulting lists of 329 **(A)** and 267 **(B)** GO terms along with their p-values were further summarized by REViGO reduction analysis tool that condenses the GO description by removing redundant terms [40]. The remaining terms after the redundancy reduction were plotted in a two dimensional space. Bubble color indicates the p-value (legend in upper right-hand corner), the two ends of the colors are red and blue, depicting lower- and higher p-values respectively. Size indicates the relative frequency of the GO term in the underlying reference EBI GOA database [41]. Bubbles of more general terms are larger.

Based on the results obtained from analysis of the effects of fold change, p-value cut off and GO level criteria, the pool of deregulated transcripts throughout all time-points was analyzed by setting the GO level at different values with the intention of obtaining more specific categories. The enrichment analysis at p-value cutoff of 10^-4^ on transcripts with a minimum of 1.5 fold changes in expression (ANOVA *t* test p ≤ 0.05) resulted in a significant reduction of the number of enriched GO terms to 329 at GO level 3 and higher. Within this collection of enriched GO terms, there are 267 terms whose GO levels are 5 and higher. The 329 and 267 terms along with their p-values were further summarized independently by the REViGO reduction analysis tool that condenses the GO description by removing redundant terms [40]. The results of these further reductions are visualized in Figures 5. Only categories with lower dispensability and frequency, i.e. more uniqueness are shown. As shown, more general terms such as adaptive and innate immune response, immune effector process, cell-cell signaling, cell communication, cell adhesion, cell activation and phagocytosis are significant at level 3 and higher (Figure 5A). Other, more specific terms are also visualized such as regulation of apoptotic process, I-kappaB kinase/NF-kappaB cascade, glial cell migration, and synaptic transmission and synapse organization. However, to achieve more specific terms, one needs to look at the terms with higher GO levels (Figure 5B). The majority of terms visualized in Figure 5B are specific terms such as mitotic cell cycle G2/M transition checkpoint, apoptotic cell clearance, hydrogen peroxide biosynthesis, signal transduction by p53 class mediator and regulation of Toll-like receptor signaling pathway.

Most enriched GO terms visualized in Figure 5 represent deregulated transcripts with the different expression profiles shown in Additional file 1: Figure S1. As expression clustering is performed prior to GO enrichment, the time-series GO enrichment may produce enriched terms that are also significantly represented by the expression profiles shown in Additional file 1: Figure S1. Indeed, we found that the majority of enriched GO terms for up-regulated transcripts represent the expression profiles 44 and 46 and in some cases, profile 48.

### Time-point GO enrichment

**Figure 6:**
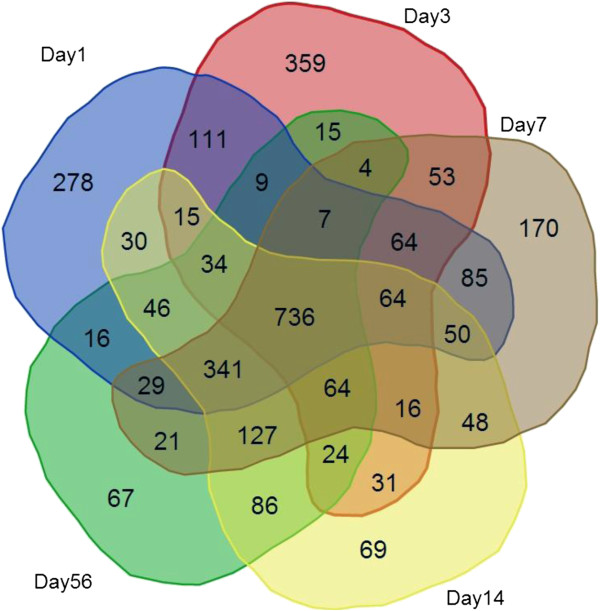
**Common and Unique GO Terms between Time-Points.** GO enrichment analysis was performed separately for deregulated transcripts (fold change ≥ 1.5, ANOVA *t* test p-value ≤ 0.05) at each time point. The enriched GO terms at a less stringent condition (p ≤ 0.05) were examined for common and unique terms using Venn diagram. All time-points were compared to each other simultaneously. Overlapping areas represent common terms between different time points. As shown, 736 terms were common to all time-points, of which 284 had a p-value ≤ 0.00001.

**Figure 7:**
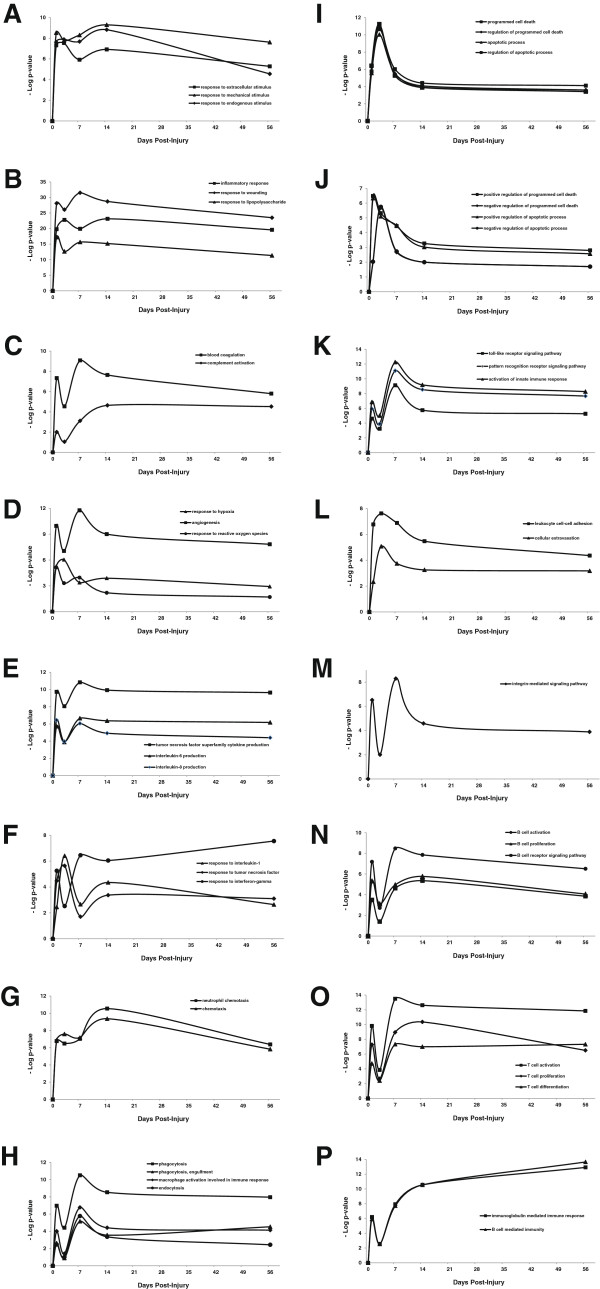
**Temporal Pattern of Various GO Biological Processes Common to all Time-Points. A-P.** Temporal pattern of change of each GO term was analyzed in order to examine the order of events after spinal cord injury. Multiple and pairwise comparisons of the enriched GO terms obtained for all time-points were made. 284 terms were found to be significantly deregulated across all time-time points post-injury (p-value ≤ 0.00001). The most specific terms were further analyzed for their gene content as well as their up- or down-regulations. The p-values of each term at various time-points were transformed to - Log10 value and plotted. Each graph depicts a single or multiple enriched GO biological process.

We next analyzed the temporal pattern of each GO term in a time-point fashion in order to examine the order of events after SCI. To accomplish this, we made multiple comparisons of the enriched GO terms obtained for deregulated transcripts (minimum fold change value ≥ 1.5; ANOVA *t* test p-value ≤ 0.05) at individual time-points. Figure 6 depicts the Venn diagram of this analysis on the multiple lists of enriched GO terms (p-value cut off of 0.05) obtained for each individual time-point. A less stringent condition was selected to allow for all possible similarities and differences to be observed. As shown, this analysis resulted in 736 common GO terms between all time-points, of which 284 had a p-value ≤ 0.00001 throughout the course of the study (data not shown). Additionally, some biological processes were shown to be significantly up- or down-regulated at only a certain time window as their respective GO terms were uniquely specified to one time point only. For example, 278, 359 and 170 terms were uniquely specified to day 1, day3 and day 7 post-injury, respectively. There are fewer unique terms detected at the chronic stage of the injury i.e. 69 and 67 for day 14 and day 56 post-injury, respectively. The fact that there are significantly higher numbers of common GO terms (i.e. 736) relative to the number of unique terms at each time-point indicates the complexity of the many common processes involved following moderate to severe SCI and that the significance of contribution of these processes is diminished within our time window. Having determined the GO levels for all categories in the previous steps, we then determined the most specific terms and positioned them in their GO tree hierarchy. A summary of significantly enriched BP terms that were found to be commonly up-regulated across all time-points is presented in Table 3. Some general terms such as the “response to external stimulus”, “response to mechanical stimulus” and “inflammatory response” possess the highest significance but belong to lower levels of GO hierarchy. Other categories, however, present more specific functions and are positioned at higher GO levels. As shown, significant up-regulation of GO terms corresponding to “response to extracellular and mechanical stimuli”, “inflammatory response” as well as “response to lipid” and “response to lipopolysaccharide” was observed across all time points, day 1-day 56 post-injury. The “angiogenesis” term is also consistently enriched in the day 1-day 56 time points. Genes involved in “blood coagulation” were up-regulated to high levels at day 1, and at day 7 through today 14. The “complement activation” process is only enriched at later time points, i.e. day 14-day 56 post-injury. Although a significant “response to hypoxia” was observed from day 1-day 3, the peak of response to “reactive oxygen species” and “hydrogen peroxide” was observed exclusively on day 1. The “response to glucocorticoid stimulus” was observed on day 1 and day 14 post-injury. “Production of IL-6” process peaked on day 1 post-injury but continued to be enriched on day 7 and at later time points, while the peaks for “tumor necrosis factor production” and “IL-8 production” were on day 7. Previous studies have shown a marked increase in TNF-alpha production immediately after injury. Since this study did not include time-points earlier than 24 hours, this result may imply the second wave of TNF-alpha production following the acute phase of the injury. Interestingly, the responses to cytokines such as tumor necrosis factor and IL-1 were observed only on day 3. “Neutrophil chemotaxis” process initiated on day 1 but peak activity was observed on day 14. From Figure 7H, it is evident that the level of “phagocytosis”-related transcripts are up-regulated early after injury but peak at one week post-injury and stay up-regulated. Likewise, the expression of genes that belong to the “Toll-like receptor signaling pathway” term alters with the same profile (Figure 7K).

**Table 3:** Common GO terms across all time-points post-injury

**Term ID**	**Parent term name**	**Term ID**	**Child term name**	**GO level**
GO:0009605	response to external stimulus	**GO:0009991**	**response to extracellular stimulus**	**4**
		**GO:0009612**	**response to mechanical stimulus**	**4**
GO:0050896	response to stimulus	**GO:0009719**	**response to endogenous stimulus**	**3**
GO:0006950	response to stress	**GO:0009611**	**response to wounding**	**4**
GO:0033993	response to lipid	**GO:0032496**	**response to lipopolysaccharide**	**7**
GO:0007599	hemostasis	**GO:0007596**	**blood coagulation**	**6**
GO:0002252	immune effector process	**GO:0006956**	**complement activation**	**4**
GO:0070482	response to oxygen levels	**GO:0001666**	**response to hypoxia**	**6**
GO:0048514	blood vessel morphogenesis	**GO:0001525**	**angiogenesis**	**11**
GO:0006979	response to oxidative stress	**GO:0000302**	**response to reactive oxygen species**	**5**
		**GO:0042542**	**response to hydrogen peroxide**	**6**
GO:0006954	inflammatory response	**GO:0002367**	**cytokine production involved in immune response**	**5**
GO:0001816	cytokine production	**GO:0042089**	**cytokine biosynthetic process**	**5,8**
GO:0001816	cytokine production	**GO:0071706**	**tumor necrosis factor superfamily cytokine production**	**5**
		**GO:0032635**	**interleukin-6 production**	**5**
		**GO:0032637**	**interleukin-8 production**	**5**
		**GO:0050663**	**cytokine secretion**	**8**
GO:0034097	response to cytokine stimulus	**GO:0070555**	**response to interleukin-1**	**6**
		**GO:0034612**	**response to tumor necrosis factor**	**6**
		**GO:0034341**	**response to interferon-gamma**	**6**
GO:0045087	innate immune response	**GO:0034341**	**response to interferon-gamma**	**6**
GO:0030595	leukocyte chemotaxis	**GO:0030593**	**neutrophil chemotaxis**	**8**
GO:0002275	myeloid cell activation involved in immune response	**GO:0002281**	**macrophage activation involved in immune response**	**7**
GO:0002274	myeloid leukocyte activation	**GO:0042116**	**macrophage activation**	**6**
GO:0012501	programmed cell death	**GO:0006915**	**apoptotic process**	**5**
GO:0006897	endocytosis	**GO:0006909**	**phagocytosis**	**7**
		**GO:0006911**	**phagocytosis, engulfment**	**8**
		**GO:0043277**	**apoptotic cell clearance**	**8**
GO:0050776	regulation of immune response	**GO:0002218**	**activation of innate immune response**	**9**
GO:0002758	innate immune response-activating signal transduction			
GO:0002429	immune response-activating cell surface receptor signaling pathway	**GO:0002220**	**innate immune response activating cell surface receptor signaling pathway**	**11**
GO:0002758	innate immune response-activating signal transduction	**GO:0002221**	**pattern recognition receptor signaling pathway**	**11**
		**GO:0002224**	**toll-like receptor signaling pathway**	**12**
GO:0016337	cell-cell adhesion	**GO:0007159**	**leukocyte cell-cell adhesion**	**5**
GO:0007165	signal transduction			
GO:0007166	cell surface receptor signaling pathway	**GO:0007229**	**integrin-mediated signaling pathway**	**7**
GO:0050900	leukocyte migration	**GO:0045123**	**cellular extravasation**	**7**
GO:0046649	lymphocyte activation	**GO:0042113**	**B cell activation**	**6**
		**GO:0042110**	**T cell activation**	**6**
GO:0046651	lymphocyte proliferation			
GO:0042113	B cell activation	**GO:0042100**	**B cell proliferation**	**7**
GO:0046651	lymphocyte proliferation			
GO:0042110	T cell activation	**GO:0042098**	**T cell proliferation**	**7**
GO:0030098	lymphocyte differentiation			
GO:0042110	T cell activation	**GO:0030217**	**T cell differentiation**	**11**
GO:0002429	immune response-activating cell surface receptor signaling pathway	**GO:0050851**	**antigen receptor-mediated signaling pathway**	**11**
GO:0050851	antigen receptor-mediated signaling pathway	**GO:0050853**	**B cell receptor signaling pathway**	**11**
GO:0002460	adaptive immune response based on somatic recombination of immune receptors built from immunoglobulin superfamily domains			
GO:0019724	B cell mediated immunity	**GO:0016064**	**immunoglobulin mediated immune response**	**7**
GO:0044707	single-multicellular organism process	**GO:0001503**	**ossification**	**4**

A significant finding is the occurrence of the “apoptotic process” on day 1-day 7 post-injury. This process is accompanied by events whose peak of response also corresponds exclusively to day 7, such as “interleukin-6 production”, “tumor necrosis factor production”, “macrophage activation involved in immune response”, “phagocytosis” and “engulfment and “apoptotic cell clearance” (Figure 7E and 7H).

On both days 1 and 7, a significant up-regulation in induced-innate immunity related GO terms such as “pattern recognition-mediated signaling’ , “Toll-like receptor signaling” and “integrin-mediated signaling pathways” was detected. “Leukocyte cell-cell adhesion” was observed from day 1 to day 7. While genes involved in activation of “innate immune response”, “B and T cell activation”, “cytokine biosynthetic process”, and “phagocytosis” were up-regulated at day 1 and from day 7 onwards to day 56; ‘T cell differentiation” and “B cell mediated immunity” up-regulation is only observed during the chronic phase of injury, i.e. day 14-day 56 (Figure 7O and 7P). Thus, it is not surprising that the “B and T cell proliferation” and the “B cell receptor signalling pathway” peaks of response were on day 7-day 14. Day 14 also marks a peak response to “ossification” (data not shown). Importantly, the peak response to interferon-gamma and the immunoglobulin-mediated immune response is observed on day 56. These two mark the late response biological processes induced after injury to spinal cord.

Our analysis also showed that “programmed cell death” and its related child terms “apoptotic process” and “positive and negative regulation of apoptotic process” are commonly enriched only during day 1- day 7 post-injury. Apoptotic processes significantly increase early after injury on day 1 post-injury and reach a peak between day 3 and day 7 post-injury, after which the contribution of apoptotic processes is diminished but stays significantly enriched (p ≤ 0.001-0.0001). Both positive and negative regulations of apoptosis are significantly enriched, which indicates the fact that the injured cells struggle for survival. However, activation of apoptosis seems to be more predominant than its suppression, as the positive regulation of apoptosis becomes activated earlier than negative regulation and its peak of activity is on day 1 post-injury, although it stays continuously up-regulated up to 1 week post-injury. In contrast, the only significant activity of negative regulation of apoptosis (p ≤ 0.00001) is on day 3 (Figure 7I-J).

We can summarize the biological processes listed in Table 3 into three main categories: stress response including processes such as blood coagulation, complement activation, response to hypoxia and reactive oxygen species, angiogenesis and inflammation. The second category consists of induced innate immune response processes such as activation of macrophages and microglia by Toll-like receptor signaling, cytokine production and secretion, chemokine production and neutrophil chemotaxis, IL6 and tumor-necrosis factor production and their responses. A significant set of induced innate immune-related biological processes involve “Phagocytosis” and “Toll-like receptor signaling pathway”. The third category is mainly the components of adaptive immune response processes such as T cell activation, migration and proliferation, B cell activation and immunoglobulin-mediated immunity. Both humoral and cell-mediated elements of “adaptive immune response” processes seem to actively participate in the pathology of SCI. The pattern of change in mRNA levels for many genes in the above GO biological processes follow the expression profiles observed in profiles 44, 46, 48 and 45, which have been discussed earlier (Figure 3A-H). The continuous up-regulation of the “immunoglobulin mediated immune response” and its parent term “B cell mediated immunity” is striking and may imply that these processes should be categorized as chronic phase responses to SCI as their peak of activity appears after 2–8 weeks, although initiated at early timepoints post-injury.

Our GO analysis also resulted in enrichment of many regulatory processes, the majority of which are positive regulations of the enriched GO biological processes listed in Table 3, as illustrated for apoptotic processes shown in Figures 7I-J.

## Discussion

### The injury model

Since its introduction in 1978 as the first SCI model in rats [20], the clip-compression model has become a standard injury model in animals as it mimics the human primary mechanism of injury to the spinal cord as well as the histo-pathological and behavioural outcomes of human SCI. Our lab has previously characterized this mode of SCI [42-44]. The clip compression of the spinal cord results in central cavitation and axonal loss in the white matter of spinal cord [45]. Rats that receive the clip-compression injury have a very similar pathological progression to humans with SCI including the formation of a cystic cavity surrounded by a glial scar [44]. In addition, animals injured by clip compression will have the same functional recovery profile as is observed in humans [20,23,28].

Previous studies have shown that the response of the spinal cord tissue to injury consists of a complex series of cellular responses and events. These cellular events are reflected in a more complex change in temporal and spatial pattern of molecular events at the mRNA level, which, in turn, depends on the type and severity of the primary injury and the following cascade of secondary events [1]. Earlier reports on high throughput gene expression analysis after SCI in animals have been almost exclusively performed in contusion-based models of injury using weight drop method [36-38,46]. As no such study on the clip-compression injury model has been reported, we aimed to investigate the rat transcriptome dynamics after a moderate to severe injury using hemorrhagic SCI by clip model, similar to most human SCIs. Additionally, the primary injury in the clip model consists of both impact and persistent compression. Therefore, we hypothesized that both similarities and differences between the two models of injury would be evident by examining how the changes in transcriptome occur. Moreover, unlike the majority of earlier studies that chiefly examined the acute and subacute events, we extended the time-frame of our study to 8 weeks post-injury to allow examination of the acute, subacute and chronic phases of the injury. The chosen time-points were based on previous behavioural and immunohistochemical analyses, which showed that following SCI by clip-compression, the first 24 hours post injury would represent a very acute stage and possible involvement of most immediate early stress genes. Days 3 and 7 represent a time during which the peak of delayed apoptotic cell death for the neural cells occurs. Days 10–14 are considered the subacute stage, as the inflammation appears to subside. Finally day 56 is considered the chronic stage as it is the time when the BBB motor recovery test for the spontaneous recovery/improvement in the rat animal model reaches a plateau.

### GO enrichment analysis as a tool for biological process inference

Functional analysis of microarray data is a challenging task as the result of initial analysis is only the fold change values representing deregulations in the expression of thousands of transcripts. There are different approaches to analyzing the results of a microarray experiment in order to make efficient biological inferences. Various platforms share a common feature in that they perform an overrepresentation analysis on the list of deregulated genes and statistically analyze if the pool of up- and/or down-regulated transcripts is significantly enriched compared to the list of genes previously annotated to be part of a defined Biological Process, Molecular Function or Cellular Component, as is the case with GO enrichment, or to a certain metabolic or signaling pathway as is observed in pathway analysis platforms. Various pathway analyses are currently in practice for microarray data analysis and there are different approaches to accomplish this. KEGG pathway [47-49], Wikipathways [50-52], and Ingenuity (http://www.ingenuity.com) are amongst the currently available platforms for pathway analysis. A recent analysis showed that among the above three pathway databases, (KEGG, Ingenuity and Wikipathways) there is a low level of consistency, comprehensiveness and compatibility [53] and the level of consistency varies significantly when different pathways are compared. Due to these limitations, and because GO is considered to represent a relatively current, comprehensive, and, more importantly, a controlled vocabulary for gene function [54], we analyzed our microarray data using GO enrichment analysis. However, we are also aware of the limitations of GO enrichment analysis [55]. For example, prior to GO enrichment analysis in this study, we determined the number of annotated genes in the list of deregulated transcripts and found that only 55% and 75% of the 14,327 genes on the Rat GeneChip 230 2.0 are annotated in the EBI and RGD association files, respectively (data not shown). The above percentages of annotated genes in Rat genome are similar to the number of annotated genes in all other organisms whose genome has been sequenced and only a subset of known genes are annotated for each of the three domains of GO tree, i.e. BP, MF and CP components [56].

An advantage in using a controlled vocabulary of gene function such as GO on the SCI microarray data comes from the challenging nature of such analysis due to the inherent complexity of the spinal cord tissue and also the type and level of injury itself. Spinal cord tissue is composed of an array of highly specialized neurons, astrocytes, oligodendrocytes, microglia, and pericytes. Another specialized and complex structure within the cord tissue whose permeability is highly compromised [57] upon injury is the blood spinal cord barrier (BSCB), which is composed of neurovascular unit (NVU), that maintains the integrity of BSCB and is again comprised of endothelial cells, neurons, astrocytes, and pericytes [58]. Additionally, SCI is generally categorized as a severe injury that leads to loss of normal physiological functions. Thus, the development of a complex series of secondary damage [1] to the spinal cord after the primary injury is due both to the vast array of cell types affected as well as the injury severity that sets many processes in motion. Such an injury model demands a nonbiased and yet comprehensive coverage of annotations such as GO for clustering of deregulated genes into relevant processes and events. The reliability of this approach is shown by its successful conjecturing of previously known biological processes as well as their dynamic of contribution to the pathology of spinal cord injury as explained below.

### Blood coagulation and blood protein signaling

The supply of blood and nutrients is crucial for normal functioning of neural cells. It is well-documented that an early and progressive development of hemorrhage is a common feature of all experimental models of SCI and this includes the clip-compression model [59,60]. Shearing of the blood vessels and disruption of the vascular architecture within the lesion epicenter by mechanical force leads to hemorrhage, a progressive process which extends to the rostral but more towards the caudal regions of the grey matter [24,61-63]. As post-traumatic ischemia develops [1,59], further vasospasm [64] and loss of autoregulation of blood flow [65,66] exacerbate the condition. Therefore, the earliest event following compression injury to the spinal cord is a profound damage to the local vasculature (capillaries and venules), hemorrhage (especially in the grey matter) and disruption of cord microcirculation by mechanical, thrombotic or vasospasm mechanisms. Consequently, the normal blood flow to the spinal cord is significantly reduced, which leads to a marked ischemia in the gray and white matter [60].

The results of our microarray data analysis clearly confirm the outcome of the primary impact and persistent compression injury to the spinal cord, which is disruption of the vasculature and hemorrhage as the major and initial result of the primary injury. Our data indicate that representative genes in the blood coagulation cascade are up-regulated (Figure 7A). For example, the transcript levels of the integral membrane protein tissue factor (coagulation factor III, F3), coagulation factors VIII (F13A1, F8), platelet factor (PF4) and V (F5) are up-regulated, the latter being elevated only on day 1 post-injury (data not shown). Permanent binding of tissue factor F3 to membrane surface is thought to be crucial for the speed of enzymatic reactions in coagulation processes [67]. Additionally, we found that platelet factor (PF4) mRNA levels were increased upon injury. PF4 (CXCL4) is a chemokine released from activated platelets to bind heparin and inhibit its anticoagulant activity. ANO6 is a transmembrane protein that may have a calcium-activated chloride channel activity but it is thought to be essential for calcium-dependent exposure of phosphatidylserine on the surface of activated platelets. Importantly, ANO6 (anoctamin 6 or TMEM16F) transcript level is also elevated early after injury and is continues to be up-regulated up to 8 weeks post-injury. Higher than normal transcript levels of ANO6 during both acute and chronic phases of SCI may explain why the coagulation process is up-regulated even at 8 weeks post-injury. Regulatory proteins such as protein C, a serine protease that is activated in the blood coagulation cascade, along with its receptor (PROCR) are up-regulated as well. Activated protein C has potent anticoagulant activity due to its ability to inactivate factor Va and VIIIa (Yesilirmak et al., 2008) and seems to alleviate the secondary SCI by reducing the ischemia/reperfusion effect by inhibiting neutrophil activation (Hirose et al., 1999) and or leukocyte activation [68], inducing insulin growth factor-1 and its receptor leading to an increased number of motor neurons [69].

The GO enrichment analysis identified another 30 coagulation-related genes whose transcripts were up-regulated throughout the course of the study. Amongst these were regulatory proteins with anticoagulant properties such as tissue factor pathway inhibitor 2 (TFPI), which is released by endothelial cells and binds factor VIIa complexes, inhibiting them to generate factor Xa. TFPI function regulates the extrinsic coagulation pathway. Additionally, we found that thrombomodulin (THBD) transcripts were elevated upon SCI up to 2 weeks post-injury. THBD binds thrombin and promotes its interaction with protein C. The resulting complex inactivates factors VIIIa and Va. Elevated levels of these regulatory proteins indicate the importance of endogenous signaling mechanisms to limit excessive spreading of clot formation.

A serious side effect of hemorrhage is the infiltration of blood components such as hemoglobin and fibrinogen to the spinal cord tissue which have been shown to be toxic to CNS tissue [70-73]. Infiltration of hemoglobin creates a hostile environment that is rich in reactive oxygen species and other toxic materials, which induces the cellular response to these toxic mediators of cell death and apoptosis. Hemoglobin, released from red cells after trauma, can promote tissue injury through iron-dependent mechanisms such as inhibiting the Na/K ATPase activity and catalyzing substantial peroxidation of CNS lipids [71]. In our study, the majority of Na/K ATPase enzymes such as ATP1A2, ATP1A3, ATP1B1 and ATP1B2 were down-regulated during the acute as well as the subacute phase of the injury (data not shown). Fibrinogen has been shown to trigger an inhibitory signal transduction pathway in neurons by acting as a ligand for beta-3 integrin, which induces the transactivation of EGF receptor (EGFR) in neurons, thereby inhibiting neurite outgrowth [73]. It also triggers astrocyte scar formation through TGF-beta signaling [72]. The microarray data in our study confirms that genes in the TGF-beta signaling cascade are up-regulated. For example, TGFB1, its receptor and SMAD2 transcripts were up-regulated throughout the 8 weeks post-injury study period (data not shown).

### Complement activation

Along with the blood coagulation cascade, a concomitant increase in the complement activation system is observed, whose temporal pattern is not the same as blood coagulation but rather develops in a more delayed fashion. The blood coagulation cascade peak of activity is on day 7 post-injury but stays up-regulated until 8 weeks. The complement activation, however, is turned on with a lag time in the first few days with activity increasing at later time points in the experiment (Figure 7C). Whether the late activation of complement system is due to the effect of reperfusion after ischemia needs further investigation. The complement system can be activated by three different but overlapping classical, lectin and alternative routes [74].

Representative genes in the complement activation system were deregulated following clip injury to spinal cord. For example, the transcript level of the main activator of the classical pathway of complement activation (C1S) is down-regulated one day after injury. However, it returns to normal values by day 3 and is further up-regulated by day 7 remaining at higher than normal levels even at day 56 post-injury (data not shown). C1S catalyzes the consecutive conversion of C4 to C4a and C4a to active C4b2a (C3 convertase), whose main function is to cleave parental C3 into C3a and C3b. As shown the mRNA levels of C1qa, C1qb, C1qc, Cfd and Cr1l are increased relative to sham un-injured animals. The transcript level of Factor H (CFH), a negative regulator of the alternative pathway for complement activation, is decreased after injury but fluctuates back to higher than normal levels by day 7 post-injury. The elevated level of CFH in our study is in agreement with previous reports that complement inhibitor proteins such as factor H were expressed at elevated levels on neurons and oligodendrocytes after SCI in rats [75,76].

Using inhibitor approaches, both classical and lectin pathways of complement activation have been shown to participate in SCI pathology [77-79]. C1q Knockout mice showed improved recovery and thus the classical complement activation via C1q is thought to be detrimental to the injured spinal cord [80]. Our data show that the mRNA level of C1 inhibitor (C1-INH, SERPING1), an inhibitor of the lectin pathway, is also increased in a similar profile as observed in CFH mRNA deregulation. C1-INH inhibits complement activation through binding and inactivating MASP1 and MASP2 [74]. Up-regulation of C1-INH has been shown to be protective and independent of C1q and the classical pathway [81].

### Ischemia, response to hypoxia and reactive oxygen species

The decrease in the local blood-flow leads to ischemic-hypoxic damage to the spinal cord tissue. Ischemia generally leads to a decrease in cytoplasmic levels of ATP, cellular swelling through malfunctioning of Na/K ATPases and also the mitochondrial membrane permeability transition [82]. Additionally, hypoxia induces certain transcription factors such as hypoxia inducible factor 1 (Hif-1) heterodimer which is composed of the inducible Hif-1a and the constitutive Hif-1b subunits [83,84]. The induction of Hif-1a is under the control of NF-kB transcription factor which serves to link hypoxia to innate immune response [85]. This is reflected in an increase in the mRNA level for the genes that function in response to hypoxia. We found that, following clip-compression injury to the spinal cord, the transcript levels of Hif-1a were up-regulated. Negative control of Hif-1 transcriptional activity is under the control of EGLN3, a propyl hydroxylase that, in the presence of oxygen molecule, permits ubiquitination and proteosomal degradation of Hif-1a monomer and Hif1an, which blocks Hif-1 transcriptional activity by preventing Hif-1 association with p300 [83-88]. In this study we found that the transcript level of EGLN3 is decreased upon injury to the spinal cord. EGLN3 acts as the cellular oxygen sensor and is the most important enzyme in promoting Hif-1a degradation. This may explain why its down-regulation causes a positive regulation of the response to hypoxia. EGLN3 has other functions such as NGF-induced proapoptotic effect in neurons, probably through regulating CASP3 activity [89].

Hif-1a induction and activation under hypoxic condition induces NF-kB and its inhibitor at the same time [83,86]. In this study, we found that NF-kB related transcripts were all up-regulated. For example, the transcript levels of NFKB2 and of the inhibitors NFKBIA, NFKBIE, NFKBIZ are all up-regulated during the first week after injury (data not shown). Another complication of disruption of blood supply is the phenomenon of ischemia/reperfusion injury causing necrotic injury to oligodendrocytes, neurons, astrocytes, and endothelial cells in the epicenter [90,91]. This involves many events such as hypoxia, reactive oxygen species (ROS) and lipid peroxidation, cytokines, complement activation, and pro- and anti-apoptotic signaling cascades [91-93]. The ischemia/reperfusion injury is mainly under the regulation of the NF-kB signaling cascade and NF-kB transcription and its signaling cascade are, in turn, responsible for positive regulation of many immune-related responses, anti-apoptotic and equally important but opposing and controversial pro-apoptotic pathways [94].

### Induced innate-immune response and Toll-like receptor signaling: a biphasic process

The inflammatory response to injury is initiated within minutes after SCI [95]. Our enrichment analysis scored inflammation as the most significant process starting immediately after injury and transcription activation of many immune-related genes. Many cytokines and chemokines are produced and secreted by various cells in the spinal cord tissue. It has been shown that IL-1B is produced immediately by astrocytes and neurons [95,96]. Similar to other studies, our data indicates an up-regulation of IL-1B and TNF-alpha after injury. Most notably, we observed that the inflammatory response, in general, and specifically the cytokines’ expression pattern follow profile 44 (Figure 3F). Profile 44 represents the change in transcript levels of many genes with the first wave of up-regulation on day 1. The early up-regulation then disappears on day 3 and comes back to high levels for many transcripts from day 7 onwards. Such a phenomenon has been reported previously in mice with a contusion injury [95], although the cessation of primary up-regulation occurred after 24 hours and returned to an increased state on day 14 post-injury. In line with this observation, a biphasic model of cellular inflammatory response has been shown when various immune cells were analyzed using flow cytometry after SCI [97]. Various categories of processes are depicted in Figure 7A-P, which confirm such studies. Accordingly, we can extrapolate our findings and assume the same mechanism of expression or secretion for transcripts with the same profile of expression. This biphasic mode of expression was observed in other enriched GO biological processes such as activation of innate immune response, response to lipopolysaccharide, response to interferon-gamma, tumor necrosis factor superfamily, cytokine production, interleukin-6 production, interleukin-8 production, cytokine secretion, neutrophil chemotaxis, endocytosis and phagocytosis Toll-like receptor signaling pathways, integrin-mediated signaling pathway, T and B cell activation, and immunoglobulin-mediated immune response. The simplest explanation for this observed biphasic response is that the first wave of transcription activation of these genes originates primarily from neurons, astrocytes and microglia cells within the injured area of spinal cord, which subside by day 3. By day 7, post-injury immune cells such as neutrophils, macrophages, T and B lymphocytes have infiltrated the injured cord and amplify the production and secretion of related cytokines and chemokines as the secondary response tends to be at a higher magnitude.

The synthesis of IL-1B in neurons was shown to be dependent on NALP1 inflammasome [96]. In astrocytes, however, overexpression of inflammatory cytokines such as CCL2, CCL3, CXCL1 and CXCL2 is triggered by the IL-1 receptor and not the Toll-like receptor signaling proteins TLR2 and TLR4. Our data indicate that the mRNA levels of IL-1B, IL-1R2 and its accessory protein IL-1RAP, were up-regulated especially on day 3. Central proteins in the Toll-like receptor signaling such as TLR2, TLR4 and MYD88 were all up-regulated, the expression pattern of which follow profile 44. It has been shown that for neutrophils to enter the damaged zone in the spinal cord, the expression of IL-1R and MYD88 are essential [98]. Additionally, the cellular extravasation of neutrophils and other leukocytes into the injured area of spinal cord also requires up-regulation of matrix metalloproteinases (MMPs) [99,100]. MMPs up-regulations are, in turn, dependent on the interaction of Fas and its ligand and on the peripheral myeloid cells and activation of Syk kinase to trigger recruitment to the injury sites [101,102]. In our injury model, we observed an increase in the mRNA levels of MMP2, MMP9 and MMP12. We did not observe an increase in transcript levels of Fas or its ligand, but the Sky mRNA was up-regulated on day 1 and afterwards up to 8 weeks post-injury.

Toll-like receptor signaling is initiated after pattern recognition receptors (PRRs) detect pathogen-associated molecular patterns (PAMPs) or danger-associated molecular patterns (DAMPs), which are endogenously generated from tissue and cellular damage. It is now thought that for induction of innate immune response, two signals are required, the first from Toll-like receptors (TLRs) and the second from Nod-like receptors (NLRs). NLRs are responsible for processing of pro-interleukin-1B to IL-1B and pro-IL-18 to IL-18 [103]. Following injury to the spinal cord, processing of pro-IL-1B and pro-IL-18 into the mature form requires NALP1, ASC (PYCARD), CASP11, and finally CASP1 action to cleave the pro- forms [96,98,104]. Activation by endogenous signals in response to SCI seems to be the mechanism of activation of inflammation after SCI. We observed the up-regulation of the NOD1 component early after SCI. We also found that, after clip-injury to the spinal cord, PYCARD and CASP1 transcripts are highly up-regulated until 8 weeks post-injury as well as IL-1B and IL-18 transcripts. In addition, the expression of purinergic receptor P2X, ligand-gated ion channel 4 (P2RX4), which has been shown to regulate the inflammasome activation after spinal cord injury [105] was persistently increased in our injury model.

### Adaptive immune response and antibody production

Both IL-1 and IL-18, produced during the first phase of inflammation mediated through the two-signal model of TLRs and NLRs, can induce the cellular and humoral modes of the adaptive immune response. IL-18 affects natural killer (NK) cells, monocytes, dendritic cells, T cells, and B cells, thereby regulating not only the innate, but also the adaptive immune responses [106]. Administration of IL-18 promotes production of interferon-gamma by natural killer (NK) as well as T cells. In our study we observe a late interferon-gamma response, which could be part of the second wave of cytokine production by T cells. T cell migration and activation precede the response to interferon-gamma, but other developing adaptive immune responses such as immunoglobulin-mediated immune response run in parallel to the response to interferon-gamma, which may explain the timing of the two processes (Figure 7).

It has been shown that autoantibodies are generated and detected in patients with chronic SCI [107,108]. These detected antibodies can recognize a variety of related and unrelated antigens to CNS tissue. Mice defective in production of B cells, and thus antibody production, exhibit reduced pathological symptoms and improved locomotor recovery [109]. The activation of B cells has been shown to be level dependent as T3 injury completely abolished B cell response and T9 injury level induces significant B cell activation and antibody production [110]. It has been postulated that delayed antibody production and accumulation of autoantibodies leads to complement activation through C1q, which triggers the enzymatic cascade of the classic complement activation pathway and recruitment of microglia and macrophages to the site of injury [111]. Our data clearly show that a delayed adaptive immune response is initiated through immunoglobulin-mediated signaling and that this response is consistently and increasingly up-regulated towards the chronic phase in parallel to activation of the complement cascade (Figure 7C and 7N-P). However, the initial events such as T cell migration, T cell and B cell activation and proliferation starts very early after the injury (Figure 7N-P). As shown, the B cell-receptor signaling pathway seems to be a much more significant process than T cell receptor signaling (Figure 7N) which implies that, compared to the cellular T-cell mediated immune response, B cell-mediated immunity and neutralizing antibody production is the dominant immune response during the chronic phase of the injury to the spinal cord (Figure 7).

## Conclusions

Microarray expression profiling was used to investigate the temporal changes in the transcriptome of the injured spinal cord in rats. Using GO enrichment analysis we show that it is possible to analyze the fold change in the expression of thousands of genes and obtain the overall picture of the processes involved. Thorough analysis of the expression profiles detected, significant biological processes and events such as response to hypoxia and reactive oxygen species were identified as early events after the injury. We found that both induced innate and adaptive immune responses are strongly and significantly up-regulated, each with relevant sub-categories and deregulated genes. The induced innate immune response may be classified as an acute to subacute type of response, whereas the adaptive immune response and antibody production can be categorized as a late response. The biphasic expression pattern identified in many genes related to immune-response implies that both resident spinal cord cell types as well as infiltrating blood cells may participate in cytokine and chemokine production and general inflammatory response. Our approach in analyzing the fold change in the mRNA levels of many deregulated genes using microarray technology indicates that with careful and systematic analysis of the data, it is possible to reliably delineate the processes involved in injury and recovery and to establish hypotheses for further analysis and intervention strategies.

## Methods

### Animal care and thoracic spinal cord injury

All experimental protocols were approved by the animal care committee of the University Health Network in accordance with the policies established in the guide to the care and use of experimental animals prepared by the Canadian Council of Animal Care. Female Wistar rats (250 g; Charles River Laboratories, 4 sham and 4 injured animals for each time point) were used for this study. Injuries by the aneurysm clip method were performed as previously described [20,29,45,112]. Briefly, under halothane anesthesia (1-2%) and a 1:1 mixture of O2/N2O, the surgical area was shaved and disinfected with 70% ethanol and betadine. A midline incision was made at the thoracic area (T4-T9), and skin and superficial muscles were retracted. Rats underwent a T6-T8 laminectomy and then received a 35 g clip (Walsh) moderate to severe compression injury at T7 for 1 min. The surgical wounds were sutured, and the animals were given Clavamox (Amoxicillin plus Clavulanic acid) for 7d and standard postoperative analgesia treatments and saline (0.9%; 5 ml) to prevent dehydration. Animals were allowed to recover and remained housed under standard condition for the duration of the experiment.

### RNA isolation, processing and microarray hybridization

Rats were sacrificed at 1, 3, 7, 14 and 56 days after injury, and a 5 mm sample of the spinal cord containing the epicenter of the injured tissue was extracted for RNA analysis. Total RNA from each individual sample was extracted using TRIzol reagent (Invitrogen, Burlington, ON, Canada). RNeasy mini spin columns (QIAGEN, Mississauga, ON, Canada) were used for purification of total RNA molecules larger than 200 bp, which excludes smaller RNAs such as miRNAs. RNA quality was assessed with a 2100 Bioanalyzer (Agilent). cRNA for microarray hybridization was prepared from 5 ug of starting RNA using the protocol supplied by Affymetrix (Santa Clara, CA). cRNA was hybridized to GeneChip Rat Genome 230 2.0 arrays (24 chips total) at the Centre for Applied Genomics, The Hospital for Sick Children, Toronto, Canada). Primary data sets were saved in a MIAME-compliant format and uploaded to GEO (series GSE45006).

### Microarray data analysis

Data analysis was performed in R with the Affy package (v1.12.2) [113] in BioConductor [114]. Data were investigated for spatial and distributional homogeneity. Normalization was performed with the sequence-specific GCRMA algorithm (package v2.6.0) in BioConductor [115]. Significance testing of this dataset was performed using linear models and pair-wise comparisons [116]. Each set of animals from a given time point was analyzed and pre-processed separately. The pre-processed data were then significance-tested using a linear modelling implemented in the limma package (v2.9.10) of BioConductor. Each sub-group was fitted to a separate factor in the design matrix, and the pair-wise contrast corresponding to differential expression of injured animals relative to control (sham) animals was extracted using a contrast matrix. Empirical Bayes moderation of the standard error [117] and false-discovery rate correction for multiple testing [118] were employed, again as implemented in the limma package. ProbeSets were deemed differentially expressed at p < 0.001 in any given comparison. Significantly different ProbeSets were visualized using the Heatplus package (v1.4.0) of Bioconductor. Euclidean distance was used as the distance metric for unsupervised hierarchical clustering using the DIANA algorithm with the cluster package (v1.11.4) in R (v2.4.1), and scaling was performed across rows. Clustering was used as a tool for replicate visualization and contrast comparison, not for gene selection [119].

The resulting gene set data with fold change and associated ANOVA *t* test p-values were analyzed by Short Time-Series Expression Miner (STEM) (discussed below), which allows the temporal expression patterns to be examined and extracted from the pool of up- and down-regulated transcripts across all time-points. Alternatively, individual time-point data were analyzed separately for up- and down-regulated genes, protein classes and signaling pathways. Both approaches were combined with functional analysis of transcripts using gene ontology (GO) enrichment.

### Time-series expression profile clustering

We used the non-parametric clustering algorithm of STEM (Short Time-series Expression Miner, version 1.3.7) that is specifically designed to analyze short time-series expression data [120]. STEM implements a novel clustering method that can differentiate between real and random patterns and clusters genes by assigning them to a series of pre-defined patterns, named expression profiles. A profile is considered significant if the number of genes assigned to it exceeds the number of genes that are expected to occur by chance. The statistical significance of the number of genes assigned to each profile versus the expected number was computed and corrected for false discovery rate at p ≤ 0.05.

### GO enrichment analysis

STEM is a statistical technique based on unsupervised clustering to find cluster-centroids followed by assignment of genes using distance-classifications, with statistical analysis using enrichment-based techniques. The biological significance of a set of genes can be assessed by GO enrichment analysis. Deregulated transcripts with ANOVA *t* test p-values ≤0.05 and fold change values > 1.5 were analyzed by the GO enrichment analysis module of STEM. Temporal analysis of the list of deregulated genes was performed using both time-series and time-point approaches. Due to more comprehensive gene coverage of RGD annotation data source file, the enrichment analysis was performed with reference to the RGD association file. For GO analysis of various expression profiles, we applied the annotations of “Biological Process” (BP) domain and the minimum expression fold change (in log_2_ scale) was set to different values from zero. Other parameters were set to different values as follows: “minimum GO level” to different values from 3 to 20, “minimum number of genes” to 5, and “multiple hypothesis correction method for actual size based enrichment” to Bonferroni. STEM also offers to run the GO enrichment analysis at different GO tree levels, which allows limiting the results to more specific terms in the directed acyclic graph (DAG) structure of the gene ontology hierarchy. In this study, the time-point GO enrichment analysis was also employed to discover common up- and down-regulated biological processes across the time-points as well as possible unique processes to each time-point. The output GO terms were used for inter-relationship analysis and visualization by Venn diagram tool and or visualized as a scatter plot or interactive graph using REViGO [40].

## Competing interests

The authors declare that they have no competing interests.

## Authors’ contributions

EF and SK designed the experiment and conducted the animal surgery and RNA sample collection. PCB analyzed the microarray data using BioConductor. SSM participated in the gene set data analysis. MC conceived the methodology for gene set data analysis, carried out the data mining, expression profiling, GO enrichment analysis, the follow up temporal analysis of the enriched terms and drafted the manuscript. MGF supervised all aspects of this work. All authors read and approved the final manuscript.

## Supplementary Material

Additional file 1: FigureS1 Time-Series Clustering of Microarray Gene Set Data Using STEM. The expression data (fold change values ≥ 1.5; p ≤ 0.05; 1 missing value allowed) 1 were loaded onto STEM platform and distinct temporal expression profiles were generated, which differentiate between real and random patterns. Profiles are numbered from 0 to 49. Each box corresponds to a model expression profile. Significant expression profiles are highlighted in color to represent a statistically significant number of genes assigned as their p-values are ordered from 0 to greater values up to 5.0E-3. The model profile is colored black while the gene expression patterns for each gene within the cluster are colored in red. Clusters with similar colors show similar patterns. To all expression profiles a zero time point was added to serve the control value (sham laminectomized animals). Genes are assigned to the most closely matching profile by statistical analysis. Significant expression profiles are highlighted in color. The X-axis represents days after injury when sampling was performed and the Y-axis denotes fold-increase or decrease in expression in log_2_ scale. Every tick mark on the Y-axis corresponds to one-log_2_ change in expression relative to sham. The filtering criterion was set to 1.5 fold (in log_2_ scale). Click here for file
