# Supplementary figures and images for: Genome-wide gene expression profiling of stress response in a spinal cord clip compression injury model
Source: BMC Genomics. 2013 Aug 28;14:583. doi: 10.1186/1471-2164-14-583 (PMC3846681; doi:10.1186/1471-2164-14-583)

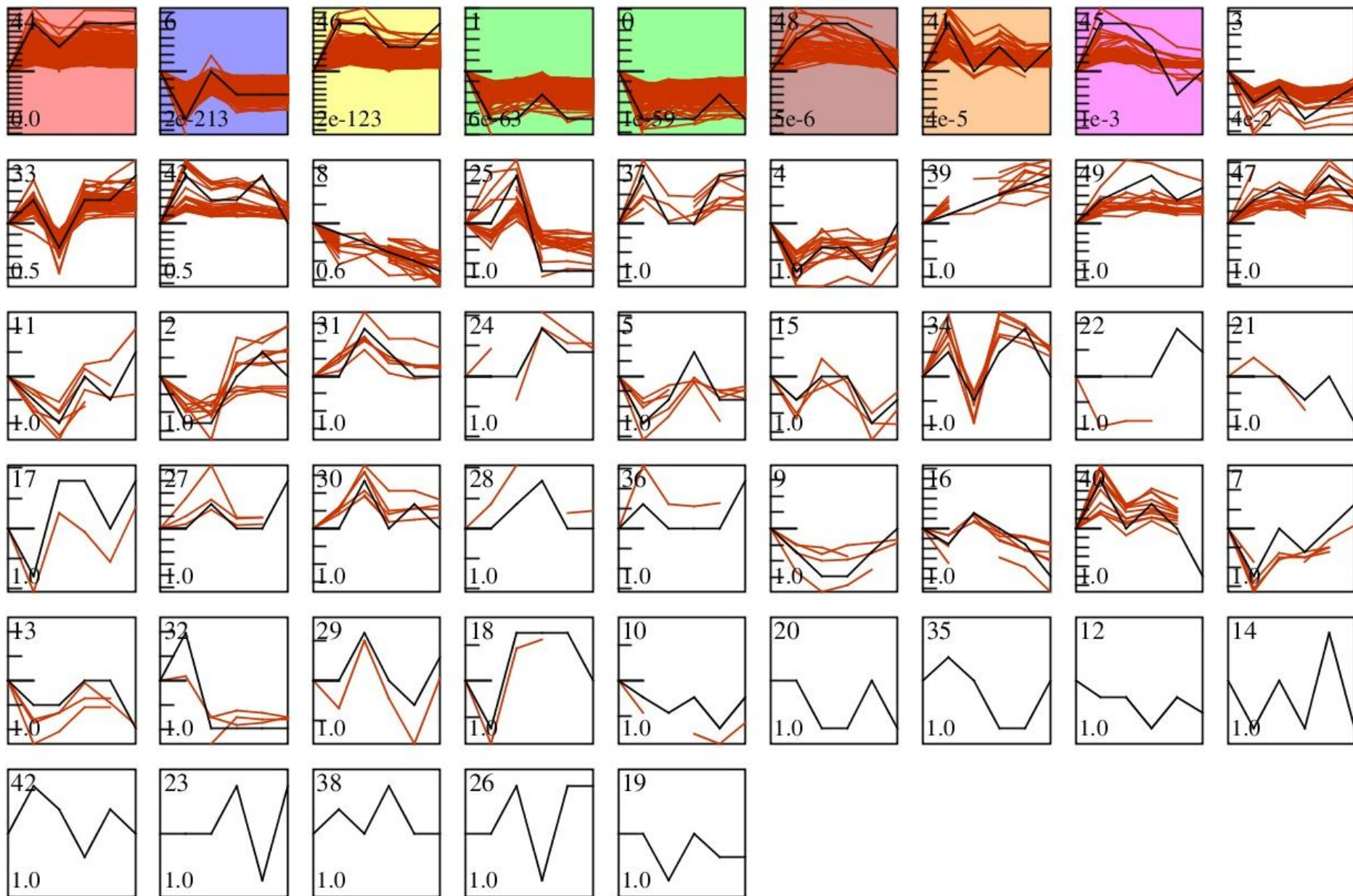

Supplement: Additional file 1: FigureS1 — Time-Series Clustering of Microarray Gene Set Data Using STEM. The expression data (fold change values ≥ 1.5; p ≤ 0.05; 1 missing value allowed) 1 were loaded onto STEM platform and distinct temporal expression profiles were generated, which differentiate between real and random patterns. Profiles are numbered from 0 to 49. Each box corresponds to a model expression profile. Significant expression profiles are highlighted in color to represent a statistically significant number of genes assigned as their p-values are ordered from 0 to greater values up to 5.0E-3. The model profile is colored black while the gene expression patterns for each gene within the cluster are colored in red. Clusters with similar colors show similar patterns. To all expression profiles a zero time point was added to serve the control value (sham laminectomized animals). Genes are assigned to the most closely matching profile by statistical analysis. Significant expression profiles are highlighted in color. The X-axis represents days after injury when sampling was performed and the Y-axis denotes fold-increase or decrease in expression in log2 scale. Every tick mark on the Y-axis corresponds to one-log2 change in expression relative to sham. The filtering criterion was set to 1.5 fold (in log2 scale). [file 1471-2164-14-583-S1.pdf]
